# Classification of Paediatric Celiac Disease Using RNA Sequencing and Real‐Time PCR of Duodenal Biomarkers

**DOI:** 10.1111/jcmm.70854

**Published:** 2025-09-22

**Authors:** Hanna Gustafsson Bragde, Sven Almer, Jan Söderman

**Affiliations:** ^1^ Laboratory Medicine Region Jönköping County Jönköping Sweden; ^2^ Department of Biomedical and Clinical Sciences Linköping University Linköping Sweden; ^3^ Department of Medicine, Solna Karolinska Institutet Stockholm Sweden; ^4^ Centre for Digestive Health, Department of Gastroenterology, Dermatology, and Rheumatology Karolinska University Hospital Stockholm Sweden

**Keywords:** celiac disease, clinical decision support, disease classification, gene expression, molecular biomarkers, RNA sequencing, RNA‐seq

## Abstract

Celiac disease (CD) diagnosis in children with sub‐threshold tissue transglutaminase autoantibody (anti‐TG2) levels requires a small intestinal biopsy. Through RNA sequencing and real‐time PCR of small intestinal biopsies, gene expression in such children was compared with the expression in children with active CD and anti‐TG2 levels above the threshold, and with non‐CD children. The study also included CD children with a non‐diagnostic first biopsy to explore early gene expression changes in CD. The aim of the study was to explore gene expression in relation to anti‐TG2 levels, investigate gene expression in Potential CD, and provide a gene expression profile to aid in CD diagnostics. The results showed that in active CD, expression changes involved genes associated with e.g., immune response, transport, angiogenesis, and epithelial barrier function, with even more pronounced changes of genes associated with cell cycle progression, absorption, lipid and lipoprotein processes, and retinoid metabolism in the active CD group with higher anti‐TG2 levels. Gene expression changes in CD children with a non‐diagnostic first biopsy showed large inter‐individual variations, but in general, gene expressions were associated with many of the same biological contexts as in active CD, including epithelial barrier function. Overall, the results show that gene expression profiling has great potential as a complement to the histopathologic assessment in CD diagnostics, even early in the disease course, but probably cannot be used for prognostic purposes.

## 1 Introduction

In recent years, the diagnostic flow of paediatric celiac disease (CD) has changed with the introduction of reliable tissue transglutaminase autoantibody (anti‐TG2) assays [1], which have led to a decrease in small intestinal biopsy samplings. There is, however, a large group still in need of a biopsy for a definite diagnosis. In a study by Wolf et al. over 50% (489 out of 898) of patients with suspected CD had anti‐TG2 values below ten times the upper limit of normal (< 10 × ULN), which is the established cut‐off level for a blood‐based diagnosis, and therefore needed a biopsy to confirm or rule out CD [2]. For parts of that group, the diagnosis is delayed because the histopathologic assessment reports a low degree of damage in the small intestine (Marsh 0–1), inconclusive for a CD diagnosis [1]. Delays can also be caused by, e.g., inadequate biopsy sampling or suboptimal orientation of the biopsy sections [3]. A delayed diagnosis is associated with reduced health status and quality of life, as well as increased levels of health care utilisation and medication [4].

Some studies have investigated the use of gene expression as a tool in CD diagnostic procedures [5, 6, 7, 8, 9, 10]. Our previous study [10] and a recently published study on formalin‐fixed, paraffin‐embedded tissue [11] indicated that changes in gene expression were present already before the pathologist could confirm changes in histology indicative of CD. In the current study, RNA sequencing (RNA‐seq) was performed on small intestinal biopsies from several groups of children with suspected or established CD enteropathy, and with varying anti‐TG2 levels.

Our current study adds knowledge by analysing gene expression associated with anti‐TG2 levels, presenting a potential gene expression profile in CD diagnostics, and investigating the possibility of using small intestinal gene expression in early CD diagnostics.

## 2 Materials and Methods

### 2.1 Study Subjects and Samples

Children and adolescents referred for small intestinal biopsy sampling for suspected CD or at follow‐up of established CD during gluten‐free diet (GFD) were included (Table 1). Most patients were referred due to elevated anti‐TG2 levels, but some for other reasons, such as a need to exclude CD or hereditary factors. Blood sampling for anti‐TG2 analysis was performed as previously described [12]. Intestinal biopsies were collected during routine upper endoscopy, and samples were collected from the bulb and the distal duodenum for diagnostic purposes, as well as from the distal duodenum for study purposes.

**TABLE 1:** Descriptive data on the celiac disease (CD) study groups.

	*n* (F)^a^	Age^b^	Marsh grade	GFD^c^	HLA‐DQ2.5cis^d^	Anti‐TG2^e^
Not CD	7 (4)	11 (4.2–16)	0	No	71%, 29%, 0%	0.029 (0–0.14)
Normalised CD	6 (5)	6.5 (4.6–17)	0	Yes	17%, 66%, 17%	0.25 (0.057–0.90)
Potential CD without progression	3 (2)	9.1 (7.2–13)	0–1	No	0%, 67%, 33%	3.3 (3.0–4.7)
Potential CD with progression	5 (4)	15 (7.3–16)	0–1	No	0%, 100%, 0%	3.4 (1.4–10)
Potential CD with high anti‐TG2 and progression	3 (3)	7.2 (3.1–15)	0–1	No	0%, 100%, 0%	15 (13–47)
Biopsy‐based CD diagnosis	6 (5)	13 (9.0–17)	3A–3C	No	0%, 83%, 17%	4.7 (2.0–8.4)
Anti‐TG2‐based CD diagnosis	6 (5)	6.5 (1.8–19)	3A–3C	No	60%, 20%, 20%	45 (18–211)
**All groups**	**36 (28)**	**9.0 (1.8–19)**	**0–3C**			**3.1 (0–211)**

*Note:* Group Not CD included children without signs of CD in the small intestine (Marsh 0) and with anti‐TG2 levels < 1 × ULN, while group Normalised CD included CD diagnosed children on a gluten‐free diet (GFD) with normalised intestine and normalised anti‐TG2 levels. Group Potential CD without progression included children who underwent biopsy on two or more occasions, with inconclusive CD histopathology (Marsh 0–1), and anti‐TG2 levels between 1 and 10 × ULN. Further, no CD diagnosis was made within the 5 years following the study biopsy, and anti‐TG2 levels normalised in most cases. Groups Potential CD with progression and Potential CD with high anti‐TG2 and progression encompassed children with inconclusive CD histopathology (Marsh 0–1) at the first biopsy and initial anti‐TG2 levels of 1–10 × ULN or > 10 × ULN, respectively, who later (within 5 years) were diagnosed with CD via biopsy. Additionally, patients with a CD diagnosis and a histopathologic assessment of Marsh 3, encompassing both those with transglutaminase autoantibody (anti‐TG2) levels 1–10 × ULN at diagnosis (group Biopsy‐based CD diagnosis), and those with anti‐TG2 levels above ten times the upper limit of normal (> 10 × ULN) at diagnosis (group Anti‐TG2‐based CD diagnosis), were included.

^a^ *n* = Total number of cases, F = number of females.

^b^ Median age at biopsy in years, including range (min–max).

^c^ Consuming a gluten‐free diet (GFD) at the time of the biopsy.

^d^ The percentages of study subjects with respectively 0, 1 or 2 HLA alpha chain DQA1*05 and beta chain DQB1*02 alleles (HLA‐DQ2.5) in *cis* are accounted for in each group.

^e^ Median (min‐max). Levels of immunoglobulin (Ig) A autoantibodies against tissue transglutaminase (anti‐TG2) in sera, expressed as times upper limit of normal (×ULN).

The study was approved by the Linköping Regional Ethical Review Board (2011/239‐31). All participants gave written, informed consent.

### 2.2 Study Groups

The study encompassed children from seven groups (Table 1): (1) Group Not CD included children without signs of CD in the small intestine (Marsh 0) and with anti‐TG2 levels below 1 × ULN, (2) Group Normalised CD consisted of children previously diagnosed with CD who, following a GFD, had both normalised histopathology (Marsh 0) and anti‐TG2 levels < 1 × ULN, (3) Group Potential CD without progression included children who underwent biopsy on two or more occasions, with inconclusive CD histopathology (Marsh 0–1), and anti‐TG2 levels between 1 and 10 × ULN. Further, no CD diagnosis was made within the 5 years following the study biopsy, and anti‐TG2 levels normalised in most cases (Table S1), (4) Group Potential CD with progression encompassed children with initial anti‐TG2 levels of 1–10 × ULN and inconclusive CD histopathology (Marsh 0–1) at the first biopsy, but who later, within 5 years, were diagnosed with CD via biopsy, (5) Group Potential CD with high anti‐TG2 and progression included children with initial anti‐TG2 levels > 10 × ULN and an inconclusive CD histopathology (Marsh 0–1) at the first biopsy, who later, within 5 years, received a biopsy‐confirmed CD diagnosis (first biopsy sampling was performed before the 2012 guidelines for the diagnosis of CD [13] had been implemented at the paediatric department of Ryhov county hospital), (6) Group Biopsy‐based CD diagnosis included children with a CD diagnosis, with anti‐TG2 levels 1–10 × ULN and a histopathologic Marsh 3 classification and (7) Group Anti‐TG2‐based CD diagnosis included children with a CD diagnosis, with anti‐TG2 levels exceeding 10 × ULN and a histopathologic Marsh 3 classification. The term “Potential CD with progression” has been taken from Ma et al. [11].

In addition, a group of three patients with Marsh 3 histopathology and negative anti‐TG2 was included in the real‐time PCR analyses only (Table S1).

### 2.3 Histopathology and Serology

Results from routine histopathologic assessments of duodenal biopsies were used, as previously described [12]. The diagnostic biopsies were classified based on the modified Marsh scale (Marsh 0, 1, 2, 3A–C) [14, 15], and the study biopsies were categorised according to the highest Marsh grade among the diagnostic biopsies for each patient. Serum levels of IgA anti‐TG2 were determined as previously described [12], using EliA kits from Thermo Fisher Scientific (Waltham, MA), with a cut‐off of 7 arbitrary U/mL (= 1 × ULN). In one patient (group Potential CD with high anti‐TG2 and progression) with selective IgA deficiency, IgG anti‐TG2 levels were used (Table S1). All other patients had IgA levels within the normal range.

### 2.4 RNA‐Seq and Real‐Time PCR

RNA was extracted from biopsies and cDNA was synthesised as previously described [9].

Libraries for RNA‐seq were constructed using Illumina TruSeq Stranded total RNA with Illumina RiboZero GOLD (Illumina, San Diego, CA) and sequenced on NovaSeq 6000 with a 2 × 151 setup. The Bcl to FastQ conversion was performed using bcl2fastq_v2.20.0.422 from the CASAVA software suite.

Gene expression analysis using real‐time PCR based on cDNA from biopsies was performed as previously described [10], with the use of Fast Advanced Master mix (Thermo Fisher Scientific) and with Applied Biosystems Taqman gene expression assays (Thermo Fisher Scientific) according to Table 2.

**TABLE 2:** Gene ID and names of two reference genes and 41 genes selected based on differential expression between celiac disease groups in the RNA sequencing analysis, including information on chromosomal position of the gene (Location), and the selected Applied Biosystems Taqman gene expression assays (Assay ID).

Gene ID	Gene name	Location	Assay ID
*UBE3A* ^a^	Ubiquitin protein ligase E3A	15q11.2	Hs00166580_m1
*ZFR* ^a^	Zinc finger RNA binding protein	5p13.3	Hs00211515_m1
*CXCL11*	C‐X‐C motif chemokine ligand 11	4q21.1	Hs00171138_m1
*CYP3A4*	Cytochrome P450 family 3 subfamily A member 4	7q22.1	Hs00430021_m1
*DDX60*	DExD/H‐box helicase 60	4q32.3	Hs01102712_m1
*E2F5*	E2F transcription factor 5	8q21.2	Hs00231092_m1
*HCP5*	HLA complex P5	6p21.33	Hs00198533_g1
*IFI27*	Interferon alpha inducible protein 27	14q32.12	Hs01086373_g1
*IFNG*	Interferon gamma	12q15	Hs00174143_m1
*IL17A*	Interleukin 17A	6p12.2	Hs00174383_m1
*MAD2L1*	Mitotic arrest deficient 2 like 1	4q27	Hs01554513_g1
*MMP12*	Matrix metallopeptidase 12	11q22.2	Hs00159178_m1
*NLRC5*	NLR family CARD domain containing 5	16q13	Hs01072123_m1
*OXCT1*	3‐oxoacid CoA‐transferase 1	5p13.1	Hs01036203_m1
*PSMB9*	Proteasome 20S subunit beta 9	6p21.32	Hs00160610_m1
*SAMD9L*	Sterile alpha motif domain containing 9 like	7q21.2	Hs00541567_s1
*APOA1*	Apolipoprotein A1	11q23.3	Hs00985000_g1
*APOC3*	Apolipoprotein C3	11q23.3	Hs00163644_m1
*CTLA4*	Cytotoxic T‐lymphocyte associated protein 4	2q33.2	Hs01011591_m1
*CXCL10*	C‐X‐C motif chemokine ligand 10	4q21.1	Hs00171042_m1
*CXCR6*	C‐X‐C motif chemokine receptor 6	3p21.31	Hs01890898_s1
*DPT*	Dermatopontin	1q24.2	Hs00355056_m1
*EAF2*	ELL associated factor 2	3q13.33	Hs00943839_m1
*GBP4*	Guanylate binding protein 4	1p22.2	Hs00925073_m1
*GBP5*	Guanylate binding protein 5	1p22.2	Hs00369472_m1
*IKZF2*	IKAROS family zinc finger 2	2q34	Hs00915979_m1
*KCNK15*	Potassium two pore domain channel subfamily K member 15	20q13.12	Hs03047113_s1
*KIR2DL4*	Killer cell immunoglobulin like receptor, two Ig domains and long cytoplasmic tail 4	19q13.42	Hs00427106_m1
*LAX1*	Lymphocyte transmembrane adaptor 1	1q32.1	Hs00214948_m1
*LCT*	Lactase	2q21.3	Hs00158722_m1
*LY96*	Lymphocyte antigen 96	8q21.11	Hs00209770_m1
*MED12L*	Mediator complex subunit 12L	3q25.1	Hs00326378_m1
*MKI67* ^b^	Marker of proliferation Ki‐67	10q26.2	Hs00606991_m1
*OCLN*	Occludin	5q13.2	Hs00170162_m1
*STAT1*	Signal transducer and activator of transcription 1	2q32.2	Hs01013996_m1
*TAP1* ^b^	Transporter 1, ATP binding cassette subfamily B member	6p21.32	Hs00388675_m1
*TNFRSF9*	TNF receptor superfamily member 9	1p36.23	Hs00155512_m1
*TNFSF13B*	TNF superfamily member 13b	13q33.3	Hs00198106_m1
*TRIM69*	Tripartite motif containing 69	15q15‐q21	Hs00298547_m1
*UBD*	Ubiquitin D	6p22.1	Hs00197374_m1
*UBE2L6*	Ubiquitin conjugating enzyme E2 L6	11q12.1	Hs01125548_m1
*UPB1*	Beta‐ureidopropionase 1	22q11.23	Hs00255472_m1
*WNT9B*	Wnt family member 9B	17q21.32	Hs00287409_m1

*Note:* Genes marked with yellow were selected as Best subset and were used in discriminant analysis.

^a^ Reference genes.

^b^ Selected using Predictor screening but not included in Best subset.

The single nucleotide polymorphism rs2187668 was used to identify the presence of the HLA alpha chain DQA1*05 and beta chain DQB1*02 alleles (HLA‐DQ2.5) on the same DNA strand (in *cis*), and was used as a measurement of the number of HLA‐DQ2.5cis alleles [10, 16] for each subject included in the RNA‐seq analysis.

### 2.5 RNA‐Seq Data Analysis

#### 2.5.1 Data Set

For analyses performed using R packages, R version 4.2.0 [17] and RStudio version 2022.12.0 Build 353.pro20 [18] were used. Unless otherwise specified, graphics were created using ggplot2 version 3.3.6 [19].

RNA‐seq based on 36 samples (Table 1) resulted in a mean of 81 million reads per sample (range 39–105). The RNA‐seq reads were aligned to genome build hg38 using STAR version 2.7.3a [20], and features (ENSEMBL id) were counted using FeatureCounts in the R package RSubread version 2.10.2 [21], with annotations (version 104) from Ensembl [22]. A mean of 62 million reads per sample mapped to exons (range 30–88). After filtering for low read counts and adding annotations using R package AnnotationDbi version 1.58.0 [23], 21,420 genes (out of 60,664; 35%) remained and were included in subsequent analyses.

#### 2.5.2 Analysis of Differential Expression and Biological Contexts

**FIGURE 1:**
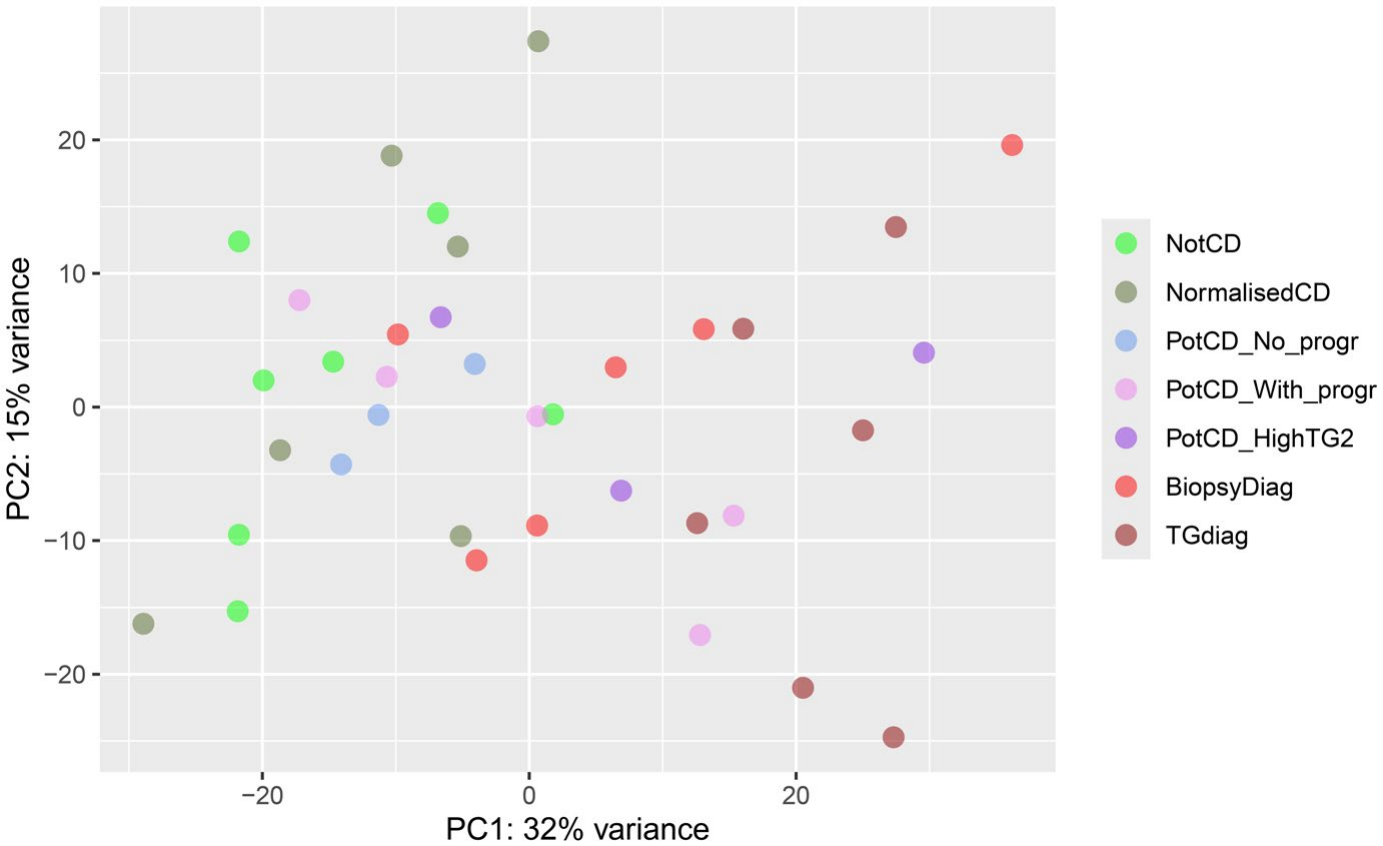
Unsupervised grouping of biopsies based on the top 1000 genes with the most variable expression from the RNA‐seq analysis, presented using PCA. The percentage of the variance accounted for by the principal component (PC) is specified for each PC. From top to bottom, colours represent groups: Not CD, Normalised CD, Potential CD without progression, Potential CD with progression, Potential CD with high anti‐TG2 and progression, Biopsy‐based CD diagnosis and Anti‐TG2 based CD diagnosis.

The R package DESeq2 version 1.36.0 [24] was used to generate normalised count data and test for differential expression between groups (Table 1), and apeglm [25] was used to shrink fold change effect sizes. The resulting differential expression data was visualised by volcano plots using R package EnhancedVolcano version 1.26.0 [26]. The shrunk fold changes were used in fast gene set enrichment analysis (FGSEA) and over‐representation analysis (ORA) to reduce effects of imprecise fold changes due to outliers and low read counts. For FGSEA [27] in the R package clusterProfiler (version 4.4.3) [28], genes were ranked based on *p*‐value and sign of the fold change. The results were filtered using the GOSemSim R package [29], where semantic similarities among enriched Gene Ontology (GO) terms were identified, and redundant GO terms removed. Additionally, clusterProfiler was used for ORA based on lists of significantly differentially expressed genes (DEGs) from the DESeq2 analysis, with a cut‐off of 0.05 for the Benjamini–Hochberg [30] adjusted *p*‐values, and an absolute log2 fold change cut‐off of 1 (based on the shrunk effect sizes). Significant GO terms were imported into Enrichmentmap version 3.3.5 [31] in Cytoscape version 3.9.1 [32] and clusters were identified using Autoannotate version 1.4.0 [33] and clusterMaker2 version 2.3.2 [34]. Unsupervised grouping of biopsies was accomplished using the top 1000 most variable gene expressions and principal component analysis (PCA) (Figure 1).

Competing statistical models were assessed using a likelihood‐ratio test in DESeq2, to investigate whether an extended model including both group affiliation (Table 1) and gender fitted the data set better than a reduced model including only group affiliation.

### 2.6 Selecting Biomarkers

Genes of interest were selected based on differential expression between groups, and biological contexts based on FGSEA and ORA were taken into consideration. Additional genes of interest from our previous publications [9, 10] were included.

In all, 29 biopsies from the RNA‐seq analysis and 70 additional biopsies were analysed for gene expression using real‐time PCR (Table S1). Correlation between real‐time PCR and RNA‐seq was investigated using the 29 biopsies.

Real‐time PCR biomarkers were identified from patients with active CD (groups Biopsy‐based CD diagnosis and Anti‐TG2‐based CD diagnosis) compared with those without CD (group Not CD), each divided into a training set and a validation set (Table S1). Based on 41 genes (Table 2), predictor screening was conducted using classification and regression decision tree methods in Statistica version 13.5.0.17 (TIBCO Software Inc., Palo Alto, CA). Optimal bins (gene groups) were identified, and their predictive power (Biopsy‐based CD diagnosis vs. Not CD and Anti‐TG2‐based CD diagnosis vs. Not CD) was estimated using the training set. The top 12 genes for each comparison were merged into a gene panel that was used as input in Best subsets identification using general discriminant analysis models (Statistica) and the Biopsy‐based CD diagnosis group training set (“Best subset”, Table 2). The selected biomarkers were then applied to the validation set using discriminant analysis to investigate their discriminatory potential using three classes (Not CD, Biopsy‐based CD diagnosis, and Anti‐TG2‐based CD diagnosis), or two classes (Not CD and Active CD).

Gene expressions from all 99 biopsies were used for PCA, visualised using the R package factoextra version 1.0.7 [35] (Figure 3).

## 3 Results

### 3.1 RNA‐Seq

#### 3.1.1 Statistical Rationale

Since the likelihood‐ratio test indicated a significant effect of gender for only 47 (0.2%) out of 21,420 genes (Table S2), all analyses were based on the simpler model without adjustment for gender.

#### 3.1.2 HLA Risk Alleles

Genotyping of the RNA‐seq study subjects using rs2187668 as a tag SNP for DQ2.5 in *cis* indicated that all children in the Biopsy diagnosis group and all of the Potential CD groups had at least one DQ2.5 in *cis*. The highest percentage of children without DQ2.5 in *cis* was found in the Not CD group, followed by the Anti‐TG2‐based CD diagnosis group (Table 1).

#### 3.1.3 Unsupervised Grouping

In the PCA (Figure 1), the Not CD group clustered to the left along the first principal component, and the Normalised CD and Potential CD without progression groups showed a tendency to skew left. In contrast, the Anti‐TG2‐based CD diagnosis group predominantly clustered to the right, a trend that was also observed in the Biopsy‐based CD diagnosis group. Potential CD with progression and Potential CD with high anti‐TG2 and progression biopsies were scattered.

#### 3.1.4 Active CD vs. Not CD

**FIGURE 2:**
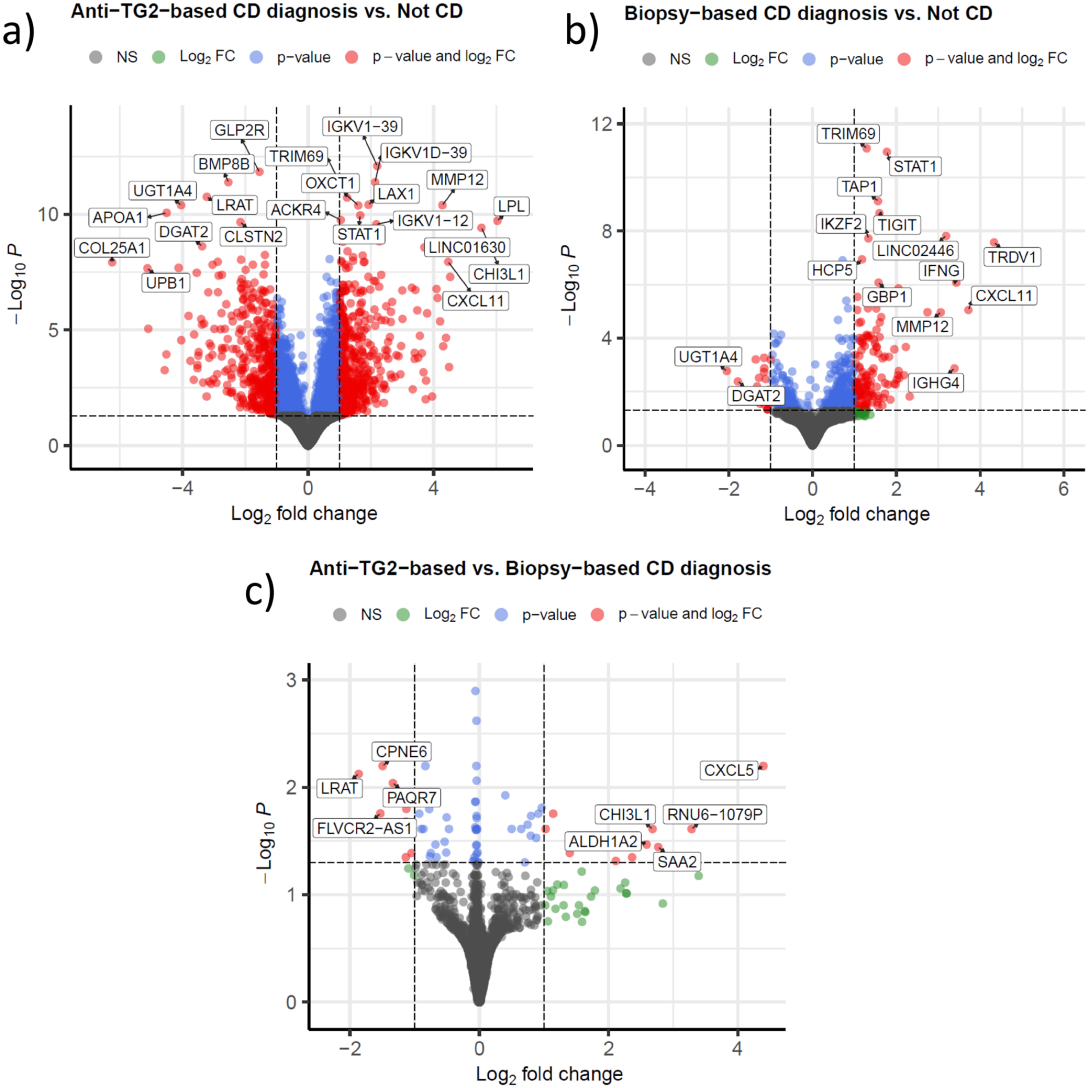
Volcano plots visualising differential expression data between groups (a) Anti‐TG2‐based CD diagnosis and Not CD, (b) Biopsy‐based CD diagnosis and Not CD, (c) Anti‐TG2‐based CD diagnosis and Biopsy‐based CD diagnosis. The y‐axis shows the Benjamini–Hochberg adjusted *p*‐values expressed as −log_10_, and the x‐axis shows the log_2_ fold changes with shrunk effect sizes. The cut‐off for a significant adjusted *p*‐value was set to 0.05, and for shrunk log_2_ fold changes a cut‐off of 1 was used. Grey markers represent genes with a non‐significant result, green markers represent genes with a significant result only for the shrunk log_2_ fold change, blue markers a significant result only for the adjusted *p*‐value, while red markers represent genes with a significant result for both.

When comparing groups Anti‐TG2‐based CD diagnosis and Not CD, 1210 DEGs with an absolute log_2_ fold change > 1 were identified, of which 878 had a shrunk absolute log_2_ fold change > 1 (Figure 2a, Table S3). ORA (Table S4) and FGSEA (Table S5) showed that genes with a higher expression in Anti‐TG2‐based CD diagnosis than in Not CD were associated with GO terms related to, e.g., adaptive and innate immunity (e.g., phagocytosis, B cell activation, regulation of lymphocyte activation, and immunoglobulin production), mitochondrial associated processes, and cell cycle progression. Genes with a lower expression in Anti‐TG2‐based CD diagnosis than in Not CD were associated with GO terms related to, e.g., transport, metabolic processes, intestinal absorption, cell junction assembly, lipid and lipoprotein associated processes, response to bone morphogenetic protein, and regulation of blood pressure and coagulation.

Between groups Biopsy‐based CD diagnosis and Not CD, 246 DEGs with an absolute log_2_ fold change > 1 were identified, of which 163 had a shrunk absolute log_2_ fold change > 1 (Figure 2b, Table S3). As in the comparison of groups Anti‐TG2‐based CD diagnosis and Not CD, ORA (Table S4) and FGSEA (Table S5) showed that genes with a higher expression in Biopsy‐based CD diagnosis than in Not CD were associated with GO terms related to e.g., adaptive and innate immunity (e.g., phagocytosis, B cell activation, MHC protein complex assembly and response to tumour necrosis factor), mitochondrial‐associated processes, and cell cycle. Genes with a lower expression in Biopsy‐based CD diagnosis than in Not CD were associated with GO terms related to transport, various metabolic processes, cell junction assembly, vascular endothelial growth factor (VEGF) and angiogenesis.

#### 3.1.5 Gene Expression Profiles Associated With Anti‐TG2 Levels

In the Anti‐TG2‐based CD diagnosis (anti‐TG2 levels > 10 × ULN) and Biopsy‐based CD diagnosis (anti‐TG2 levels 1‐10 x ULN) groups, the Marsh grades were all of grade 3A–3C. A total of 54 DEGs with an absolute log2 fold change > 1 were identified between the groups, of which 23 had a shrunk absolute log2 fold change > 1 (Figure 2c, Table S3). Based on these 23 genes, five significantly enriched GO terms were found using ORA (Table S4). One term concerned response to vitamin A, and the other four concerned metabolic processes, with the most specific term being retinoid metabolic process. FGSEA (Table S5) showed that genes with a higher expression in the Anti‐TG2‐based CD diagnosis group were involved in e.g., complement activation, phagocytosis, humoral immune response, B cell activation, rRNA metabolic processes, mitochondrial gene expression, and response to unfolded proteins. The Anti‐TG2‐based CD diagnosis group had a lower expression of genes associated with e.g., MHC class II antigen presentation, regulation of natural killer cell mediated immunity, response to interferon‐alpha, metabolic (e.g., fatty acid, xenobiotic) and catabolic (e.g., oligosaccharide, lipid) processes, intestinal absorption, and lipid and lipoprotein processes.

#### 3.1.6 Low Anti‐TG2 Levels and No or Mild Enteropathy

There were seven genes in the RNA‐seq dataset with significant differential expression between biopsies from the Potential CD with progression and the Potential CD without progression groups: *CD55*, *ARL14*, *RHPN2*, *LINC02446*, *GBP4*, *C6orf141* and *CXCL11* (Table S3). No DEGs were found between the Potential CD groups with progression and anti‐TG2 levels over or under 10 × ULN.

Genes associated with GO terms concerning e.g., different immune‐related contexts and proliferation were expressed at a higher level in the Potential CD group with progression than in the group without progression, while genes with a lower expression in the group with progression were associated with e.g., regulation of haemostasis, transport, blood circulation, metabolic processes, and intestinal absorption (Table S5).

The Potential CD without progression group clustered close to the Not CD group in the PCA (Figure 1). There were only five DEGs between these two groups, *CD55*, *ARL14*, *RHPN2, SLC7A11* and *TRDV1* (Table S3). FGSEA showed that genes with a higher expression in Potential CD without progression were included in GO terms concerning lymphocyte‐mediated immunity, phagocytosis, mitochondrial‐associated processes and endoplasmic reticulum stress (Table S5). A lower gene expression in the Potential CD without progression group was associated with, e.g., protein prenylation, cilium organisation, and homophilic cell adhesion via plasma membrane adhesion molecules.

### 3.2 Real‐Time PCR Versus RNA‐Seq

The real‐time PCR dataset included 43 genes (Table 2). Two genes were used as reference (*UBE3A* and *ZFR*) and showed a stable expression between groups and replicates in the current RNA‐seq dataset (data not shown). *KCNK15* and *WNT9B* expressions were barely detected and thus excluded from the real‐time PCR dataset. Data from RNA‐seq and real‐time PCR performed on RNA from the same set of biopsies (*n* = 29) showed a strong correlation, with a mean Pearson correlation coefficient of 0.89 (0.63–0.98) (Table S6).

### 3.3 Selecting Biomarkers for CD

**FIGURE 3:**
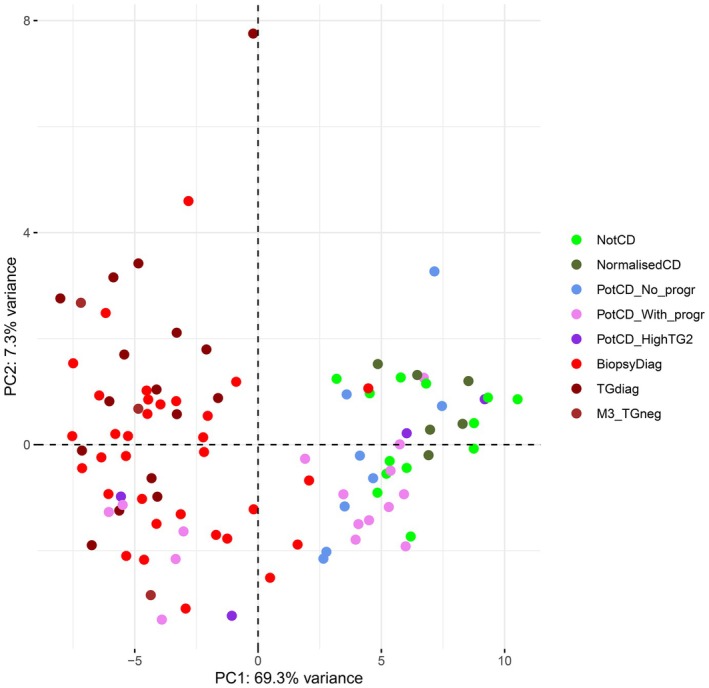
Unsupervised grouping of biopsies based on gene expression real‐time PCR data from the 14 genes selected as CD biomarkers, presented using PCA. The percentage of the variance accounted for by the principal component (PC) is specified for each PC. The marker from group Anti‐TG2‐based CD diagnosis at the top of the plot is the misclassified biopsy from case 55, while the marker from group Biopsy‐based CD diagnosis that clusters within the Not CD group is the misclassified biopsy from case 51. From top to bottom, colours represent groups: Not CD, Normalised CD, Potential CD without progression, Potential CD with progression, Potential CD with high anti‐TG2 and progression, Biopsy‐based CD diagnosis, Anti‐TG2 based CD diagnosis, and Marsh 3 histopathology and negative anti‐TG2.

**FIGURE 4:**
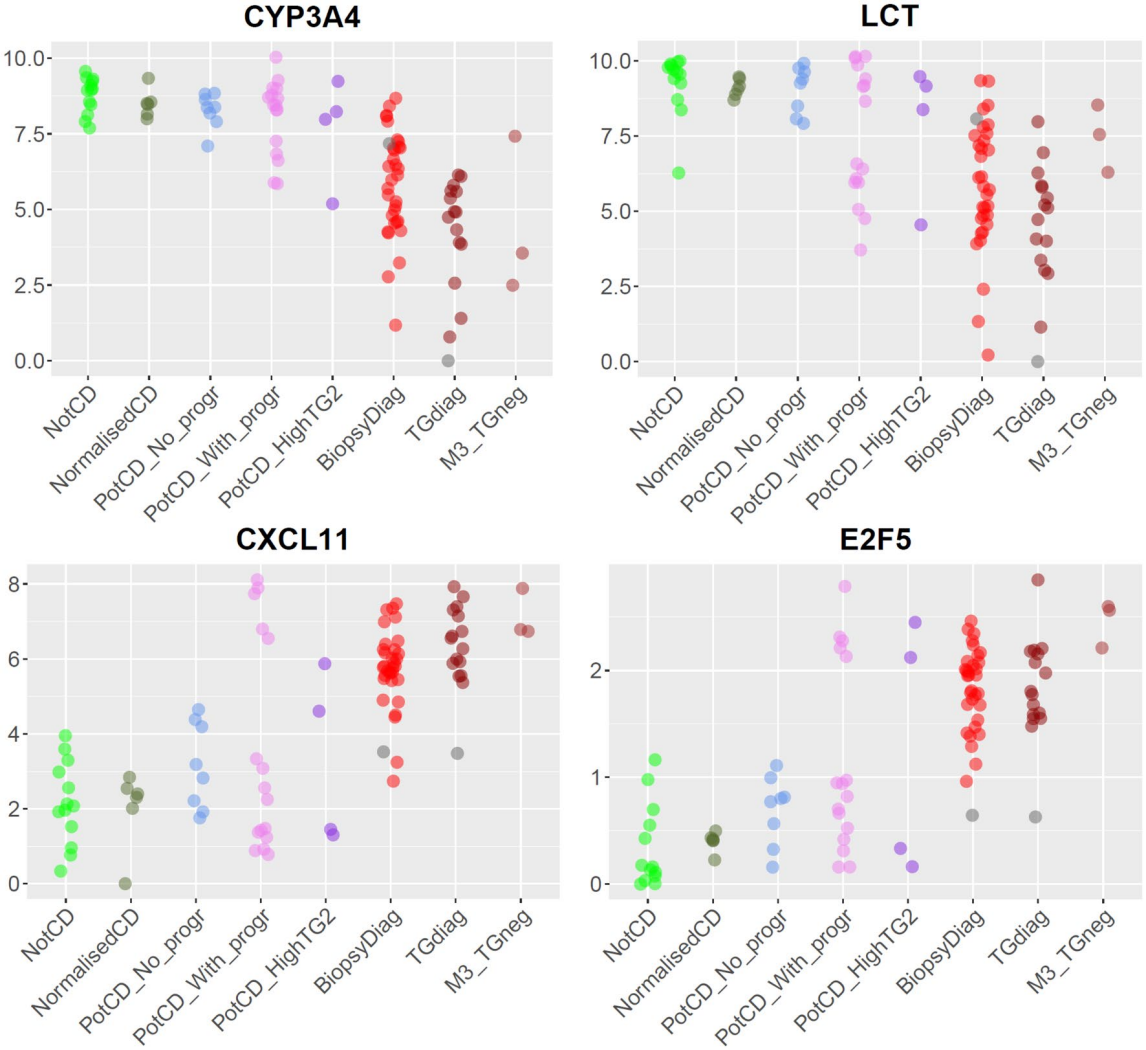
(a–d) Expression of (a) *CYP3A4*, (b) *LCT*, (c) *CXCL11*, and (d) *E2F5*, normalised to the lowest expression in the whole dataset and presented on a log_2_ scale. The grey marker in group BiopsyDiag represents the misclassified biopsy from case 51, and the grey marker in group TGdiag represents the misclassified biopsy from case 55. From left to right in each plot, the groups are: Not CD, Normalised CD, Potential CD without progression, Potential CD with progression, Potential CD with high anti‐TG2 and progression, Biopsy‐based CD diagnosis, Anti‐TG2 based CD diagnosis, and Marsh 3 histopathology and negative anti‐TG2.

For biopsies in the group Potential CD without progression, gene expression was more similar to the Not CD group than to the active CD groups, while for the Potential CD with progression group, there were large differences in gene expression among biopsies. In the RNA‐seq based PCA (Figure 1), the group with progression was scattered. This variation was also found using real‐time PCR (Figures 3 and 4a–d). We therefore sought to find genes with the ability to distinguish between active CD with Marsh grade 3 and Not CD, and investigate how those genes classify Potential CD with progression biopsies. Predictor screening based on the training sets for groups Biopsy‐based CD diagnosis and Anti‐TG2‐based CD diagnosis compared with Not CD identified 16 candidate genes for sample classification, and 14 of these were selected using the Best subsets method for discriminating between Biopsy‐based CD diagnosis and Not CD (Table 2). The fourteen selected genes were *CXCL11*, *CYP3A4*, *DDX60*, *E2F5*, *HCP5*, *IFI27*, *IFNG*, *IL17A*, *MAD2L1*, *MMP12*, *NLRC5*, *OXCT1*, *PSMB9* and *SAMD9L*.

### 3.4 Classification of Biopsies Based on Selected Biomarkers

The selected 14 genes showed 100% specificity for active CD in the validation sets, regardless of whether a diagnosis had been established at the time of the biopsy or not (Table 3) [36]. The sensitivity for established CD (Biopsy‐based CD diagnosis, Anti‐TG2‐based CD diagnosis, and Marsh 3 histopathology and negative anti‐TG2) was 93%, and decreased when Potential CD with progression and Potential CD without progression were included (77%) (Table 3).

**TABLE 3:** Diagnostic test performance for the discriminant analysis based on the 14 selected celiac disease (CD) biomarkers.

	Sensitivity (%)	Specificity (%)	PPV	NPV
Set 1	92.6	100	100	91.4
Set 2	76.6	100	100	77.1

*Note:* Set 1 includes validation set biopsies from the established active CD groups (Biopsy‐based CD diagnosis, Anti‐TG2‐based CD diagnosis, and Marsh 3 histopathology and negative anti‐TG2) and the established non‐CD group and the normalised CD group (Not CD and Normalised CD). Set 2 additionally includes biopsies from the Potential CD groups with and without progression. Positive predictive value (PPV) and Negative predictive value (NPV) were calculated based on a 56% prevalence of CD in paediatric patients referred for small intestinal biopsy sampling for suspected CD, who agreed to be part of our study (*n* = 197).

## 4 Discussion

The current study demonstrates that there are differences in intestinal gene expression between paediatric CD patients with different anti‐TG2 levels, and that the proposed expression profile of 14 genes has potential as a tool in CD diagnostics. We [9, 10], and others [5, 6, 7, 8, 37], have previously investigated gene expression profiling to characterise the small intestinal status in CD. This approach seems to have great potential as a complement to the histopathologic assessment in CD diagnostics, even if our study indicates that gene expression profiling has less prospect as a prognostic tool.

### 4.1 Gene Expression Profiles Associated With Anti‐TG2 Levels

In active CD patients, gene expression differed depending on whether anti‐TG2 levels were above (group Anti‐TG2‐based CD diagnosis) or below (group Biopsy‐based CD diagnosis) the recommended cut‐off (10 × ULN, [1]). Since these two groups were similar with respect to Marsh grade and gender, these differences potentially reflect disease processes after the Marsh 3 grade enteropathy has been established (explored in more detail in the sections below).

#### 4.1.1 Immune Response

The increased expression of B cell activation associated genes in both the Anti‐TG2‐based CD diagnosis and the Biopsy‐based CD diagnosis groups compared with the Not CD group is in agreement with the involvement of B cells in the pathogenesis of CD [38]. In Anti‐TG2‐based CD diagnosis, there were elevated levels of genes associated with both B cell activation, B cell receptor signalling pathway, and immunoglobulin production compared with Biopsy‐based CD diagnosis, possibly reflecting an increased activation of TG2‐specific B cells in patients with anti‐TG2 levels above the cut‐off compared with patients with levels below it.

#### 4.1.2 Cell Proliferation and Absorption

Genes associated with the cell cycle were more expressed in the Biopsy‐based CD diagnosis and Anti‐TG2‐based CD diagnosis groups than in the Not CD group, with the highest expression again found in Anti‐TG2‐based CD diagnosis. This increase could lead to a disturbed ratio between differentiated and undifferentiated cells, and is in agreement with the hyperproliferation of epithelial cells found in CD [39]. Potentially, changes in cell cycle progression could have implications for epithelial shedding. Amundsen et al. propose an interesting model, where shed epithelial cells from the intestine release TG2 for interaction with gliadin in the lumen, creating TG2:gluten complexes that potentially could be taken up by TG2‐specific B cells localised in gut‐associated lymphoid structures [38].

Absorptive enterocytes are present along the villus axis, with enterocytes that absorb lipids predominantly located at the very tip of the villi [40]. In line with this, the Anti‐TG2‐based CD diagnosis group showed a decrease in gene expression related to intestinal absorption, and additionally, genes associated with lipid and lipoprotein processes were lower expressed both in the Anti‐TG2‐based CD diagnosis and Biopsy‐based CD diagnosis groups than in the Not CD group, with the lowest expression found in the Anti‐TG2‐based CD diagnosis group.

#### 4.1.3 Transglutaminase

For TG2 (gene *TGM2*) there were no differences in RNA expression levels between any of the groups, indicating that the higher levels of anti‐TG2 in group Anti‐TG2‐based CD diagnosis are not a consequence of a general increase in *TGM2* expression in the small intestine. TG2 is mostly found in the cytosol, but can also be found associated with the cell membrane, and in the extracellular matrix [41]. The internalisation of TG2 from the cell membrane is cholesterol‐dependent [41], so the decrease in lipid‐associated processes, as indicated by gene expressions, may interfere with this process. This could lead to more membrane‐bound TG2 being available for interactions. However, studies indicate that activation of B cells through TG2‐gliadin complexes involves the extracellular form of TG2, and not the membrane‐bound form [42]. The proposed release of TG2 by shed epithelial cells in the model by Amundsen et al. [38] would make a better candidate. The increase in gene expression associated with cell cycle progression was especially high in Anti‐TG2‐based CD diagnosis in our study, so the formation of TG2:gluten complexes could then be augmented in this group.

#### 4.1.4 Retinoids

In the ORA, most of the gene groups identified as differentially expressed between the Anti‐TG2‐based CD diagnosis and Biopsy‐based CD diagnosis groups were linked to retinoid metabolism and response to vitamin A, and in the FGSEA, genes associated with the retinol metabolic process were less expressed in Anti‐TG2‐based CD diagnosis than in Biopsy‐based CD diagnosis. The metabolism of retinoids in the enterocyte is dependent on *LRAT* and possibly *DGAT1* for re‐esterification, before being packed into chylomicrons together with cholesterol and triglycerides and secreted into the lymphatic system for systemic uptake [43]. This process may be affected by the decreased expression of *LRAT* and of genes involved in lipoprotein‐associated processes found in Anti‐TG2‐based CD diagnosis compared with Biopsy‐based CD diagnosis. Deficiency in vitamin A may lead to problems with vision, immunity, and reproduction, and vitamin A is further needed for the maintenance of differentiated epithelial tissues [43], which is affected in CD.

#### 4.1.5 Angiogenesis

Specific for the biopsy‐based CD diagnosis group was a decreased expression of genes related to VEGF production. The mucosal vasculature in the small intestine of active CD patients is altered in organisation, as well as in the number and maturity of vessels, and anti‐TG2 inhibits angiogenesis in vitro [44]. The decreased expression of VEGF production‐related genes could play a role in the vascular alterations found in active CD.

#### 4.1.6 Epithelial Barrier Function

Genes related to cell junction assembly were expressed less in the Biopsy‐based CD diagnosis and Anti‐TG2‐based CD diagnosis groups than in the Not CD group. This expression difference was also found between Potential CD with and without progression. A decrease in cell junction assembly possibly results in an increase in paracellular permeability and influx of pathogenic gliadin peptides into the lamina propria [45]. In addition, anti‐TG2 can modulate epithelial barrier function [46].

### 4.2 Diagnostic Test Performance

The discriminant analysis based on the 14 CD biomarkers was 100% specific in regard to active CD, meaning that no non‐CD or normalised CD was misclassified as having active CD. The sensitivity was somewhat lower (92.6%), where two biopsies from the active CD groups were misclassified as Not CD. Patchy enteropathy could be an explanation for misclassifications, as indicated by the fact that biopsies from the same patient and time point showed distinct expression profiles correlating with either non‐CD or active CD (Figures 3 and 4a–d). Other reasons could include technical issues during sampling (leading to e.g., incomplete biopsies), or that a duodenal biopsy was misclassified as a bulb biopsy. The bulb is subjected to gastric acid and has a somewhat different cellular composition compared with the distal duodenum [47]. How this affects the expression of the 14 genes is not known.

The inclusion of the Potential CD groups with and without progression kept the specificity at 100% but decreased the sensitivity from 93% to 77%. This shows that the gene expression profile correctly identifies the Potential CD without progression biopsies as non‐CD but performs poorly in the Potential CD with progression group.

The 14 genes do not completely distinguish between Biopsy‐based CD diagnosis and Anti‐TG2‐based CD diagnosis, but as long as no treatment recommendations are based on these subgroups, a classification based on two classes (Active CD and Not CD) is sufficient. Regarding CD enteropathy, two classes (Active CD and Not CD) and three classes (Biopsy‐based CD diagnosis, Anti‐TG2‐based CD diagnosis, and Not CD) had the same discriminatory potential (Table S1).

In the group with Marsh 3 histopathology and negative anti‐TG2, two patients were young (< 1 year), and so anti‐TG2 production may be less efficient [48], and the third patient was older (15 years). All three had IgA levels within the normal range, and they were classified as Anti‐TG2‐based CD diagnosis in the discriminant analysis, which indicates that gene expression in this group was similar to that of Anti‐TG2‐based CD diagnosis, despite low anti‐TG2 levels.

The association between HLA‐DQ2/8 and anti‐TG2 levels is not completely elucidated [49, 50]. We did not perform an entire HLA‐DQ typing, but the tagging SNP for HLA‐DQ2.5 in *cis*, analysed in the RNA‐seq study subjects, indicated that there was not an overrepresentation of HLA‐DQ2.5 in the Anti‐TG2‐based CD diagnosis group compared with the Biopsy‐based CD diagnosis group.

### 4.3 Low Anti‐TG2 Levels and No or Mild Enteropathy

The biological contexts identified when comparing gene expression in the Potential CD groups with and without progression were similar to those found when comparing active CD with Not CD, including a decreased expression of cell junction assembly genes. In the study by Ma et al. the similarities in gene expression between active CD and Potential CD with progression in the general biological contexts were identified for the upregulated genes, but not the downregulated ones [11]. The gene expression is, however, heterogeneous in our Potential CD with progression group. Wolf et al. [51] suggested that genes that need to react quickly to changes in the environment, such as immune response‐related genes, have a high expression variance. The heterogeneity in gene expression observed in the Potential CD with progression group may be associated with initial differences in the cellular and tissue environment prior to the onset of inflammatory disease and reflected in the substantial variation in expression of CD biomarker genes such as *IFNG*, *CXCL11*, and *MMP12* (Table S1). Another possible explanation is that the Potential CD with progression group is partly comprised of patients with a patchy enteropathy, which has been found in 59% of paediatric CD patients [52]. In addition to recommended diagnostic methods [1], a gene expression profile based on RNA from intestinal biopsies, such as the one suggested in the present study, could be used to characterise a particular group of patients more likely to later develop CD. Consequently, biopsy sampling at multiple time points and a delayed diagnosis could be avoided. Other methods have been proposed, such as risk assessment methods for developing villous atrophy [53], or the use of methods capable of investigating larger parts of the small intestine [52].

**FIGURE 5:**
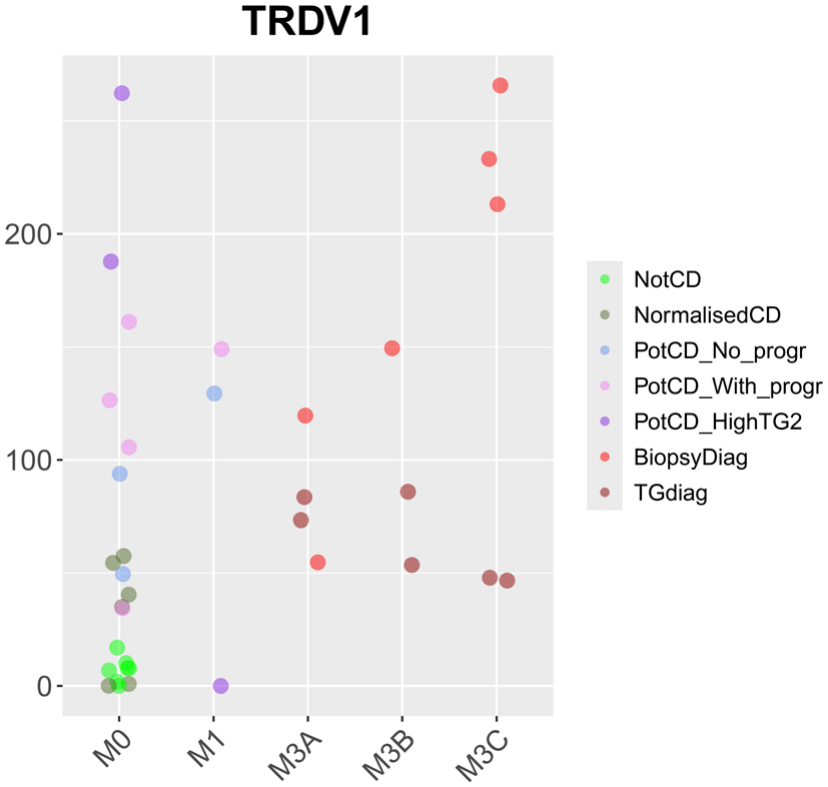
Expression of gene *TRDV1* in the RNA‐seq analysis, presented per Marsh grade (M). From top to bottom, colours represent groups: Not CD, Normalised CD, Potential CD without progression, Potential CD with progression, Potential CD with high anti‐TG2 and progression, Biopsy‐based CD diagnosis, and Anti‐TG2 based CD diagnosis.

*TRDV1* (T Cell Receptor Delta Variable 1) was expressed to a higher degree in the Potential CD without progression group, as well as in all active CD groups, than in the Not CD group (Table S3). CD patients have an increased frequency of intraepithelial γδ T cells, and the most prevalently used γδ T cell receptor V gene in the gut is *TRDV1* [54]. The increased expression of *TRDV1* in Potential CD with and without progression, Biopsy‐based CD diagnosis, and Anti‐TG2‐based CD diagnosis compared with that in Not CD could, hence, reflect an increased frequency of intraepithelial γδ T cells and may serve to measure intraepithelial γδ T cell infiltration in the mucosa. When comparing Marsh 0 biopsies in the Not CD group with biopsies from all other groups, *TRDV1* RNA‐seq expression levels were elevated in all Marsh 3 biopsies, all Marsh 1 biopsies except for one (from the Potential CD with high anti‐TG2 and progression group), and in most of the Marsh 0 biopsies (Figure 5). γδ T cells are involved in maintaining the integrity of the epithelial barrier, and it is hypothesised that they have a role in regulating the cytotoxic activity of αβ T cells [55]. γδ T cell frequency has been found to be elevated also in mucosa from patients with positive CD serology but normal mucosa [55].

### 4.4 CD Biomarkers

Several of the selected genes for the expression profile are involved in immune response‐related processes, including *DDX60*, *IFNG*, *NLRC5*, *PSMB9*, *SAMD9L*, *IFI27*, *CXCL11*, *MMP12* and *IL17A*. All showed a higher expression in active CD than in Not CD. In a study by Dotsenko et al. IFNG and genes associated with the epithelial interferon‐γ response were major contributors to gene expression changes in response to a gluten challenge in individuals with CD, a response which was prevented by the administration of a TG2 inhibitor [56]. In our study, *IFNG* showed a higher expression in both the Biopsy‐based and the Anti‐TG2‐based CD diagnosis groups compared with Not CD, but there was no significant difference in expression between the two CD groups. Further, the long noncoding RNA gene *HCP5*, located within the HLA class I region, is involved in immunity and plays a role in autoimmune diseases and cancer [57]. The drug metabolising enzyme CYP3A4 is expressed in the villi tips [58], and therefore a potential marker for villous atrophy. *MAD2L1* is associated with cell proliferation [59] and is expressed in the crypts [60], and might be a marker for crypt hyperplasia. *E2F5* belongs to the E2F family of transcription factors, the members of which regulate cell functions related to the cell cycle and apoptosis [61]. The protein encoded by *OXCT1* is involved in ketone body metabolism [62], and the use of ketonic bodies as an energy source in CD is supported by the presence of high levels of 3‐hydroxybutyric acid found in the blood of adult CD patients [63].

We acknowledge that the size of some of the groups in this study is rather small. This could have implications for the generalisability of the results, so further studies may be needed. Also, the use of separate biopsies for histopathology and gene expression analysis is a potential source of variation. Furthermore, the expression heterogeneity in the Potential CD with progression group indicates that there may be subgroups within the group that we, using the data at hand, cannot distinguish between, even though we have replicate biopsies at different time points. The Potential CD groups need to be further investigated to determine a way to establish a diagnosis as early as possible.

## 5 Conclusions

Taken together, CD (as reflected by gene expression) involves, among others, an induction of the immune response and increases in cell cycle progression, and decreases in intestinal absorption, lipid and lipoprotein associated processes, transport, and retinoid metabolism. This is in generally in agreement with previous studies of the CD transcriptome [5, 6, 7, 37, 64]. In addition, we found that for some genes, the expression in Marsh 3 CD biopsies from children with high anti‐TG2 levels (above 10xULN) differed from that in children with lower anti‐TG2 levels. Pathways associated with these genes included e.g., B cell activation and signalling, cell cycle progression, retinoid metabolism, lipid and lipoprotein processes, and angiogenesis. For many of the pathways, both groups showed a difference compared with the Not CD group, but also compared to each other. The proposed gene expression profile with 14 genes could be used in the assessment of small intestinal status in the context of suspected CD and during follow‐up on GFD. The number of genes included in such a profile may vary, and so may the selection of genes. Other genes and profiles have been suggested for the same purpose [5, 6, 7, 8, 9, 10]. A major consideration in the selection of a gene set should be that the genes reflect different processes that occur in the small intestine in CD. The potential of a gene expression profile as a prognostic tool in CD diagnostics is less clear.

## Author Contributions

**Hanna Gustafsson Bragde:** conceptualization (lead), data curation (lead), formal analysis (equal), funding acquisition (equal), investigation (lead), methodology (lead), project administration (lead), resources (lead), software (lead), supervision (lead), validation (lead), visualization (lead), writing – original draft (lead), writing – review and editing (equal). **Sven Almer:** data curation (supporting), formal analysis (supporting), resources (supporting), visualization (supporting), writing – original draft (supporting), writing – review and editing (equal). **Jan Söderman:** conceptualization (supporting), data curation (supporting), formal analysis (equal), funding acquisition (equal), investigation (supporting), methodology (supporting), resources (supporting), software (supporting), validation (supporting), visualization (supporting), writing – original draft (supporting), writing – review and editing (equal).

## Conflicts of Interest

The authors declare no conflicts of interest.

## Supporting information


**Table S1:** Descriptive statistics on study subjects included in Discriminant analysis (DA), including the results from the DA. Additionally, a visualisation of gene expressions from the genes included in Best subset has been provided.

**Table S2:** Results from the comparison of two different models for the DESeq2 analysis; one model that included only group affiliation as a coefficient, and one that included group affiliation and gender. Presenting the genes for which the likelihood‐ratio test in DESeq2 indicated a significant effect of gender.

**Table S3:** Results from the analysis of differential gene expression using DESeq2.

**Table S4:** Results from over representation analysis.

**Table S5:** Results from fast gene set enrichment analysis.

**Table S6:** Results from the correlation of RNA‐seq data with real‐time PCR data using RNA from the same biopsies.

## Data Availability

The data that support the findings of this study are available on request from the corresponding author. The data are not publicly available due to privacy or ethical restrictions.
